# Machine Learning-Based Human Posture Identification from Point Cloud Data Acquisitioned by FMCW Millimetre-Wave Radar

**DOI:** 10.3390/s23167208

**Published:** 2023-08-16

**Authors:** Guangcheng Zhang, Shenchen Li, Kai Zhang, Yueh-Jaw Lin

**Affiliations:** 1 School of Mechanical Engineering, University of Shanghai for Science and Technology, Shanghai 200093, China; g.c.zhang@usst.edu.cn (G.Z.); 212171601@st.usst.edu.cn (S.L.); z232191370@163.com (K.Z.); 2 College of Engineering and Engineering Technology, Northern Illinois University, DeKalb, IL 60115, USA

**Keywords:** human posture, FMCW millimetre-wave radar, machine learning, comprehensive evaluation

## Abstract

Human posture recognition technology is widely used in the fields of healthcare, human-computer interaction, and sports. The use of a Frequency-Modulated Continuous Wave (FMCW) millimetre-wave (MMW) radar sensor in measuring human posture characteristics data is of great significance because of its robust and strong recognition capabilities. This paper demonstrates how human posture characteristics data are measured, classified, and identified using FMCW techniques. First of all, the characteristics data of human posture is measured with the MMW radar sensors. Secondly, the point cloud data for human posture is generated, considering both the dynamic and static features of the reflected signal from the human body, which not only greatly reduces the environmental noise but also strengthens the reflection of the detected target. Lastly, six different machine learning models are applied for posture classification based on the generated point cloud data. To comparatively evaluate the proper model for point cloud data classification procedure—in addition to using the traditional index—the Kappa index was introduced to eliminate the effect due to the uncontrollable imbalance of the sampling data. These results support our conclusion that among the six machine learning algorithms implemented in this paper, the multi-layer perceptron (MLP) method is regarded as the most promising classifier.

## 1. Introduction

Human postures can visually convey information about the human body, which finds applications in various fields such as safety production, human vital signs monitoring, and information interaction. As society embraces informatization, accurately detecting and classifying human body postures can yield effective responses in recognition systems. For instance, in coal mines’ underground operations, where working conditions can be extremely dangerous, identifying human body targets more effectively can reduce accidents [1]. Moreover, aging and accidental falls heavily impact the physical function of the elderly, leading to severe injuries. Real-time posture recognition of the elderly enables timely assistance and prevents falls [2]. In the domain of human-computer interaction, human body postures act as information carriers, serving as valuable data for recognition systems [3].

With technology advancements, various methods for detecting human body postures are emerging. Vision-based systems utilize cameras to capture human postures, extract features from contours, and employ recognition algorithms for posture recognition [4,5,6]. However, concerns over privacy limit the acceptance of cameras at home or work [7], and vision-based systems may suffer performance limitations during hostile weather conditions. Alternatively, wearable devices are used for posture detection, but they can be inconvenient and costly [8]. Radar-based systems, on the other hand, offer a non-intrusive solution that addresses privacy concerns and remains robust under different lighting conditions. This system utilizes radio waves to determine the position of a target by processing the echo signal.

Due to its ability to obtain position information of target and static objects, along with observing their slight vibrations and speeds, FMCW radio technology is widely used in many scenarios. For example, in the field of measurement, Wang et al. [9] proposed a calibration method using millimetre wave radar and camera fusion. Compared with the traditional calibration method, the calibration error is reduced significantly. Other applications include automotive radar [10], drone detection [11], snow research [12], contactless measurement [13,14], vital sign detection [15], remote sensing [16], gait recognition [17], etc. With the increasing demand for healthcare technology, surveillance, and human-machine interfaces, the use of FMCW mmWave radar to recognize human postures has become an important topic. He et al. [18] proposed using FMCW radar for human target recognition in non-direct sight scenes. The experimental results demonstrated accurate identification of real humans and human-mimicking man-made objects, even in blocked scenes. Zhang et al. [19] developed a multi-angle entropy feature and an improved ELM method for identifying human activity. The experiment achieved over 86% accuracy for outdoor scenes and 98% for indoor micro-movements. Aman Shrestha et al. [20] introduced a method based on recurrent long and short-term memory (LSTM) and bi-directional LSTM network architecture for continuous human activity monitoring and classification, achieving an average accuracy of over 90% when combined with Doppler domain data from FMCW radar. Liang et al. [21] designed a fall detection system based on FMCW radar, using Bi-LSTM for classification. The system achieved a remarkable 99% classification accuracy. Zhou et al. [22] presented a method for human sleep posture recognition based on MMW radar. The radar echo signal was processed to obtain multi-channel 2D radar features, and neural networks were employed for learning and classification. The results effectively distinguished different sleeping postures. Overall, the use of FMCW mmWave radar for recognizing human postures continues to be an important area of research and development.

Currently, 3D target recognition is a significant area of research. Point cloud data, which includes 3D coordinates (x, y, z), density, reflection intensity, and other features, offers more information than images. Huang et al. [23] used point clouds for 3D face model reconstruction to aid identification. Wang et al. [24] established a 3D mining area model using point cloud data for environmental analysis. Poux et al. [25] utilized point clouds for indoor 3D modeling and object classification. Point clouds are also widely used for generating and classifying human postures. Zhao et al. [26] proposed a human tracking and identification system (mID1) based on FMCW millimeter-wave radar, achieving an overall recognition accuracy of 89% among 12 individuals and 73% intruder detection accuracy. Meng et al. [27] developed mmGaitNet, a deep learning-driven millimeter-wave gait recognition method with 90% accuracy for a single-person scenario. Aiujaim et al. [28] used FMCW radar to recognize multiple human activities, classifying motion with an 80% accuracy based on point cloud data. While single neural network models offer automated feature extraction, machine learning is more suitable for this study due to the high data volume and computational complexity [29,30,31]. Diraco et al. [32] achieved a 97% classification accuracy using the SVM algorithm on 3D human posture point clouds. Werghi et al. [33] employed a Bayesian classification model based on wavelet transform coefficients, achieving 98% accuracy. However, the above studies only use a single machine learning model and do not put forward a comprehensive method for evaluating the classification effect of machine learning.

This paper aims to evaluate the performance of different machine learning models on human posture point cloud data based on FMCW mmWave radar. This goal is achieved in two stages. In the first stage, the characteristics data of human posture are measured and collected using the radar sensor and then the human body posture point cloud data is generated considering both the dynamic and static features of the reflected signal for the human body. Based on the previous research method [34], six hundred sets of point cloud posture data were obtained from one hundred sets of point cloud data for each of the six postures (hands up, horse stance, lunge, lying down, standing, and sitting). In the second stage, the point cloud dataset is used for six machine learning classification models, namely K-nearest neighbor (KNN), Gaussian process (GP), SVM, multi-layer perceptron (MLP), naive Bayes (NB), and gradient boosting (GB). Finally, a comprehensive performance evaluation for the different machine learning models is conducted.

The contributions of this paper are summarized as follows:

(1) This paper presents the application of FMCW millimetre-wave radar in multiple human body posture characteristics data measurements. The experiment shows that it can reflect the posture characteristics of the human body effectively.
(2) To delete the non-interesting reflection points and realize the grouping of objects from the generated point cloud data, the clustering technique (DBSCAN algorithm) is introduced to traverse all the points in the space based on the density characteristics of the point cloud distribution.
(3) To achieve feature importance ranking, Gini index-based random forest algorithm is utilized to obtain the normalized contribution of the feature, and further sort the feature according to the size of the contribution.
(4) To avoid the side effects from the uneven number of samples and compare the classification performance of different machine learning models, the Kappa Index is included along with other traditional evaluation criteria to evaluate the classification performance based on the proposed signal processing methods.

The rest of this paper is organized as follows. Data collection and processing is described in Section 2. Section 3 represents the proposed classification research methodology. Section 4 presents the results and analysis, and Section 5 concludes the paper.

## 2. Data Collection and Processing

In this paper, taking advantage of the simple system structure, simple signal processing, and low cost, the linear frequency modulated continuous wave is selected as the signal of millimetre-wave radar for generating chirp signals. During collecting and processing the data, the signals are transmitted by the transmitting antenna and reflected after encountering the target. The receiving antenna receives the reflected frequency modulation pulse and then mixes with the local transmitting signal for amplification and filtering processing. Finally, sampling and digital-to-analog conversion are carried out to obtain the original matrix data of signal processing.

### 2.1. Data Collection

**Figure 1:**
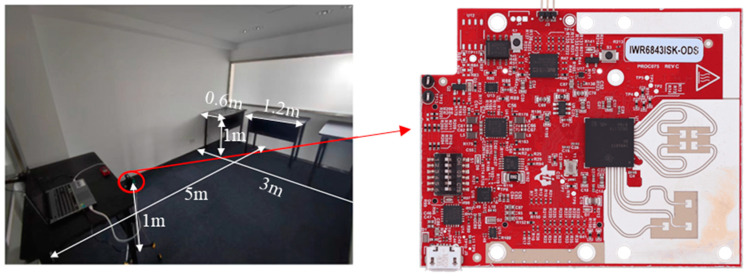
Experimental environment and layout.

**Figure 2:**
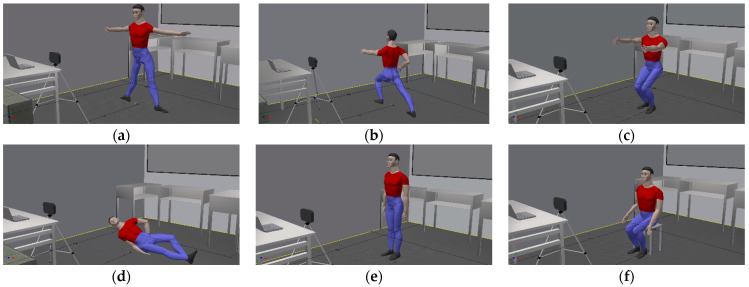
Six postures for the data collection: (**a**) hands up, (**b**) lunge, (**c**) horse stance, (**d**) lying down, (**e**) standing and (**f**) sitting.

A peaceful and clutter-free workspace is necessary for the experiment’s accuracy. An office has been chosen as the experimental scene, and no other items are in the office except for desks and experimental devices. The interior dimensions and layout of items have been illustrated in Figure 1. The size of the room is 3.1 m × 4.9 m, and the radar is located at the center of the left side of the office, approximately 1 m above the floor. On the right side of the office, there are three desks evenly spaced, each measuring 1.2 m × 0.6 m × 1 m. The height of the experimenter is 175 cm, weighs 75 kg, and is 1.5 m away from the radar. The subject then faces the radar in six different postures, known as hands up, horse stance, lunges, lying down, standing, and sitting, as shown in Figure 2.

To collect the raw radar data and perform subsequent processing, this paper uses the TI IWR6843ISK-ODS millimetre-wave sensor as the experimental device. The operating frequency is 60 GHz and there are four receiving antennas and three transmitting antennas, with 120° azimuth and 120° elevation angle coverage. The specific radar parameter configuration is listed in Table 1. For each posture, 20 s were captured with 100 frames of data per posture. The DCA1000 evaluation module is used to provide real-time data capture and streaming for radar sensors. The computer reads and processes the raw data captured by the evaluation module.

### 2.2. Data Processing

**Figure 3:**
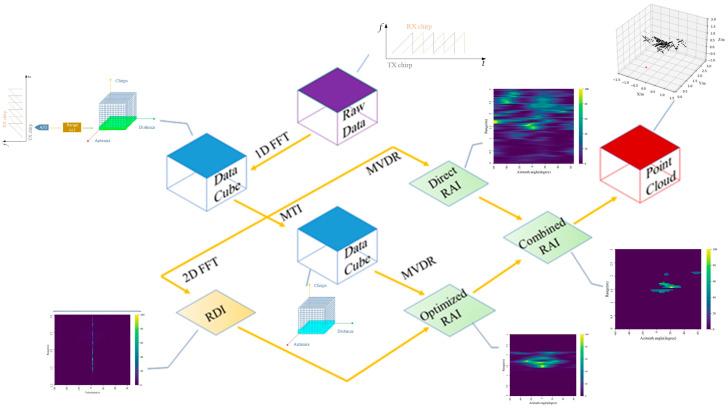
Data processing flow.

First, the Range fast Fourier transform (FFT) is employed on the raw radar data to obtain the target range information. In order to remove static clutter in the signal, a moving target indication (MTI) algorithm is applied. Second, Range Doppler Images (RDIs) are introduced to reduce multipath reflection noise in MTI results. The direct Range Angle Images (RAIs) are obtained from the results of the Range FFT with the help of the minimum variance undistorted response (MVDR) angle estimation algorithm combined with the RAIs. After MTI and MVDR, the more detailed features of the direct RAIs are located and extracted, and finally, the combined RAIs are used to generate point clouds. The specific processing steps are illustrated in Figure 3.

(1) Introduction to Methods of Processing Data Usage: In processing raw radar data, FFT (Range FFT and Doppler FFT), MVDR angle measurement, and MTI are used. The FFT method is often used in radar signal processing but will not be elaborated here.

The MVDR is a commonly used digital beamforming algorithm, and its essence is spatial filtering. It employs a beam with a certain shape to selectively pass the target signal, while the interference signal is suppressed to a certain extent. There are two types of beamforming: analog and digital, among which digital beamforming is the main method of spatial filtering. This paper assumes that the receive antenna is an array of *N*, and the received signal of the receive antenna is *S_r_*(*t*); the signal received by the array can be expressed as:

(1) $$x\left(t\right)=S_{r}\left(t\right)*a\left(\theta \right)$$

where $a\left(\theta \right)$ = ${\left[1,e^{j^{\frac{2\pi d\sin\left(\theta \right)}{\lambda }}},\ldots ,e^{j\left(\left(N-1\right)\frac{2\pi d\sin\left(\theta \right)}{\lambda }\right)}\right]}^{T}$.

The output power at different angles is calculated as follows:

(2) $$P_{mvdr}=\frac{1}{a{\left(\theta \right)}^{H}R^{-1}a\left(\theta \right)}$$

where $R=x_{t}*x_{t}^{H}$, the angle value of the targets can be obtained.

Moving target indication (MTI) is a technology for extracting moving targets from radar reflected signals. Their premise is that the reflection value of the stationary object is stable, while the reflection signal value of the moving object changes with the change of the object’s distance from the sensor position. After the Range FFT, chirps in the frame obtain frequencies corresponding to their respective distances. The distance of stationary targets remains constant within a frame and the distance of moving targets varies within a frame. Therefore, when considering the Range FFT results of all chirp signals in a frame, the chirp vector at each distance corresponds to the centering process. This means that the chirp vector at each distance is subtracted from the mean of the chirp vector. The method of processing the chirp signal is as follows:

(3) $$DI=\sum_{i=1}^{n}\left(FFT\left(i\_chirp\right)-\sum_{i=1}^{n}\frac{FFT\left(i\_chirp\right)}{n}\right)$$

where $FFT\left(i\_chirp\right)$ represents the Range FFT result for the *i*-th chirp, and *n* represents that there are *n* chirp signals in each frame of data.

(2) Acquisition of Combined RAI: The traditional radar signal processing is to perform Range FFT on the AD sampled data for each chirp to gain the distance information of the target. After the Range FFT, Doppler FFT is applied to the chirp signal at each location, and the speed information of the target is obtained.

**Figure 4:**
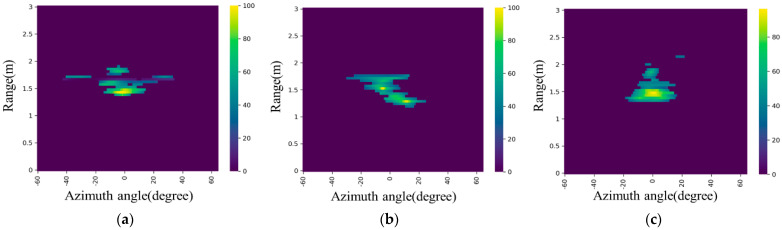

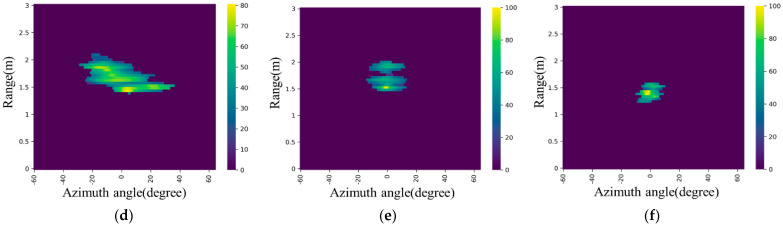
Combined RAI: (**a**) hands up, (**b**) lunge, (**c**) horse stance, (**d**) lying down, (**e**) standing, and (**f**) sitting.

However, it is difficult to include multiple target information contemporaneously. This paper presents the acquisition method of the human’s range-angle image, which includes the human’s distance, angle, and reflection intensity. MTI is used to eliminate static clutter. In order to remove the multipath reflection noise, the doppler information in the RDI is fused based on the RAI. Seen as the numerical value in the RAI based on the MTI refers to the intensity of the movement and not the reflection intensity of the static human postures, the original human reflection can be obtained by performing the MVDR algorithm on the data after Range FFT and the combined RAI can be obtained which includes the reflection intensity of human posture and remove multipath noise and static clutter. The combined RAIs are shown in Figure 4.

(3) Generate Point Clouds: After obtaining the Combined RAIs of the six postures to express the posture features more clearly and intuitively, a constant false alarm rate (CFAR) algorithm is used to generate the human target point cloud based on the RAI of two different planes. CFAR shows that the false alarm rate of the detection performance of the radar system is kept at a certain value [35]. This is a detection algorithm that guarantees the performance of radar detection and is used for point cloud detection.

**Figure 5:**
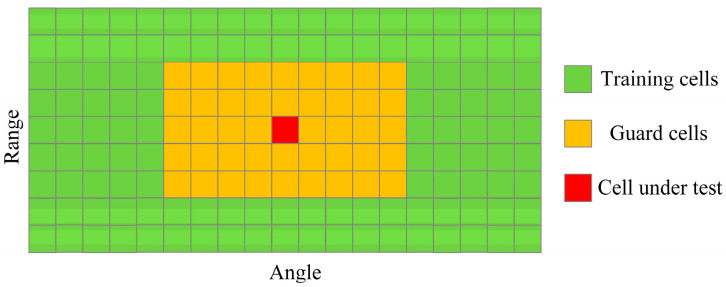
2D-CFAR peak detection.

In order to apply this algorithm to the combined RAI, this paper introduces the 2D-CFAR algorithm. The algorithm divides data cells into three types during detection: training cells, guard cells, and cells under test, as shown in Figure 5. A certain range of guard cells is set near the cell under test to prevent energy leakage that may lead to a high threshold and affect judgment. Outside the guard cells are the training cells, and the mean value of the training cells is used as the detection threshold. The value of the cell under test is compared with the threshold to determine whether there is a target point in the cell under test. Through this algorithm, the human target points can be separated from the combined RAI.

Each RAI contains the reflected power values of the target at different distances and angles (horizontal angle or pitch angle). To obtain the spatial 3D point cloud of human posture, it is necessary to fuse the reflected power values of human posture on two angular planes at different distances. Namely, the range, azimuth, and elevation angle of the target point need to be determined. Assume that the peak list of the RAI obtained from the azimuth angle direction is represented by the set $H_{1}^{*}=\left\{P\left(range,\;azimuth\;angle,\;power\right)\right\}$, including the range, azimuth angle, and human reflected power. $H_{2}^{*}=\left\{P\left(range,\;elevation\;angle,\;power\right)\right\}$ represents the peak list of the RAI in elevation angle direction, including the range, elevation angle, and human reflected power. Correlate the points of the two planes with the distance value and the power value to obtain the point set of the target three-dimensional space point cloud, the generation method is shown in Equation (4), where ⊕ represents a fusion of the data of the two planes [34].

(4) $$H_{1}^{*}⊕H_{2}^{*}⟶\{P_{\left(range,\;azimuth\;angle,\;elevation\;angle,\;power\right)}\}$$

**Figure 6:**
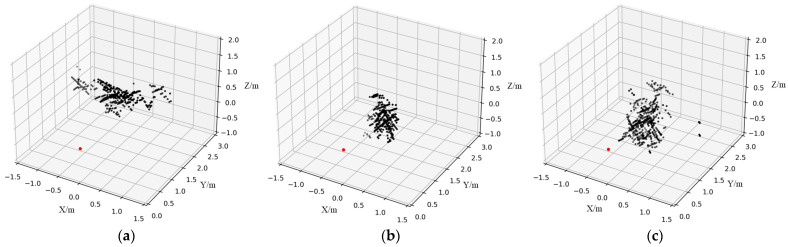

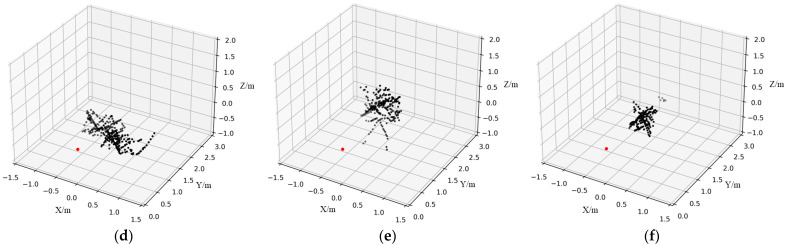
Point cloud of six postures: (**a**) hands up, (**b**) lunge, (**c**) horse stance, (**d**) lying down, (**e**) standing, and (**f**) sitting.

The six posture point cloud images obtained by this method are shown in Figure 6.

## 3. The Proposed Classification Research Method

**Figure 7:**
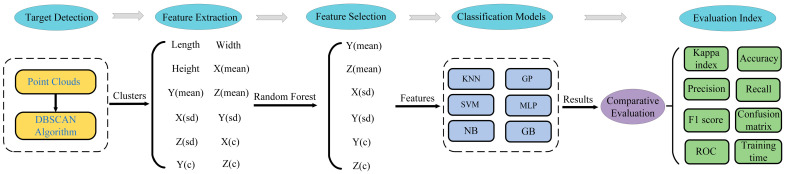
Research method flow.

The flow of the research method for human posture classification based on FMCW millimetre-wave radar proposed in this paper is shown in Figure 7. There are three key parts, namely object detection with the Density-Based Noise Applied Spatial Clustering (DBSCAN) algorithm, feature extraction, and posture classification. The classification uses six different supervised machine learning models, namely KNN, GP, SVM, MLP, NB, and GB.

### 3.1. Target Detection

**Figure 8:**
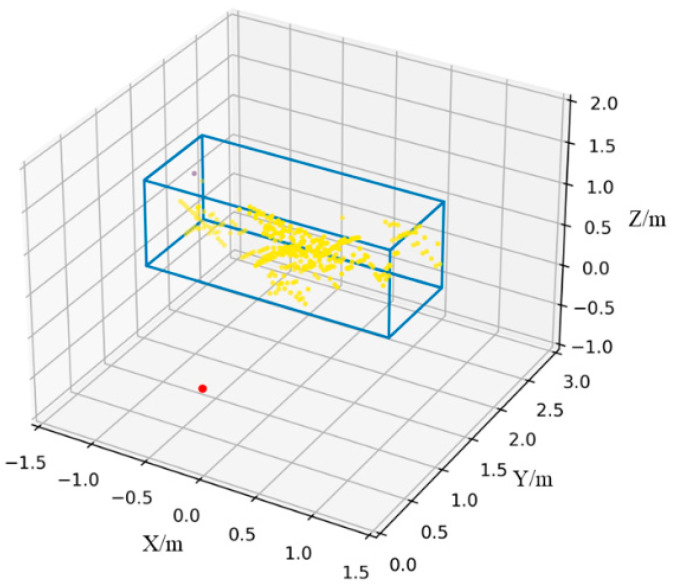
Result of DBSCAN algorithm applied to large character posture point cloud.

The CFAR algorithm detects the RAI in both angular planes and matches the detected values that are beyond the power threshold to yield point clouds. Point clouds can be denoted as a set of four-dimensional points:

(5) $$p=\{p_{i}=(x\_i,y\_i,z\_i,{power}_{i})|i=1,2,\ldots ,n\}$$

where *n* represents the number of points in the point cloud, each point contains (x, y, z)-coordinates information and reflected power. Nevertheless, the CFAR algorithm tends to return reflection points that are not targets of interest resulting in false alarms. When the DBSCAN algorithm is used, all points that correspond to the same item of interest can be grouped and non-interesting reflection points can be deleted. DBSCAN is a density-based spatial clustering technique. All the points in the space can be traversed using the density characteristics of the point cloud distribution, and the peak points can be divided to realize the grouping of objects. A point is centered on itself and Eps as the radius, if the circle contains more points than Minpts, the point is considered a core point. If the number of points contained is fewer compared to MinPts, the point is defined as a border point. An outlier point is one that is neither a core point nor a border point. If a point P is in the Eps neighborhood of the core point Q, the object P has been referred to as being directly density reachable from the object Q. A density cluster is formed by a core point Q and all objects whose density is reachable [36]. The radius Eps and the threshold of the number of items in the neighborhood MinPts are the two input parameters for the method. In this study, Eps was set to 0.5 and MinPts was set to 20. The result of clustering has been demonstrated in Figure 8. The point cloud splits the human posture into a cluster, and there is no noise in the result.

### 3.2. Feature Extraction

Feature extraction in this research can be divided into two parts, the former is feature extraction, and the latter is feature selection.

(1) Extract features: After human posture is detected, a set of interesting reflection points is obtained, which is called the human posture point cloud. Different from the point cloud generated by other methods, the point cloud generated by millimetre-wave radar integrates the information of range, angle, and reflection power of the target, which can accurately reflect the morphological characteristics of the target. Additionally, further processing is required to extract information for each posture. This section recommends that twelve features taken from human posture point clouds and radiation intensity be used to characterize the posture type, and that posture classification can be performed using these features. Table 2 shows the symbols and brief descriptions of the twelve-point cloud features. The following is a detailed description of each of the suggested features. The geometry of the point cloud for the six human postures varies widely. Therefore, this paper proposes using a rectangular box to represent the shape of the posture. The rectangular box has three dimensions: length, width, and height, which correspond to the *x*-, *y*-, and *z*-axis values respectively. Thus, this paper defines the first, second, as well as third object features F0, F1, and F2 as the difference between the maximum value and the minimum value on the *x*, *y*, and *z*-axis, namely the length (*L*), width (*W*), and height (*H*) of the rectangular box, the calculation formula is expressed as:

(6) $$F0:L=\max\left(X\right)-m\mathrm{in}\left(X\right)$$

(7) $$F1:W=\max\left(Y\right)-\min\left(Y\right)$$

(8) $$F2:H=\max\left(Z\right)-\min\left(Z\right)$$

where X represents the *x*-axis coordinate value of all object points, Y represents the *y*-axis coordinate value of all target points, and Z is the *z*-axis coordinate value of all object points.

The mean value of each posture on the three-dimensional coordinates is different. Therefore, the fourth, fifth, and sixth object features, F3, F4, and F5, are defined as the mean values on the *x*-axis, *y*-axis, and *z*-axis, which are represented by $X_{mean}$, $Y_{mean}$, and $Z_{mean}$. The calculation formula is expressed as:

(9) $$F3:X_{mean}=mean\left(X\right)$$

(10) $$F4:Y_{mean}=mean\left(Y\right)$$

(11) $$F5:Z_{mean}=mean\left(Z\right)$$

Similarly, the standard deviation of each posture on the three-dimensional coordinates are defined as the 7-th, 8-th, and 9-th features, which are represented by $X_{sd}$, $Y_{sd}$, and $Z_{sd}$. The calculation formula is expressed as:

(12) $$F6:X_{sd}=\sqrt{\sum_{i=1}^{n}{\left(X_{i}-X_{mean}\right)}^{2}/n}$$

(13) $$F7:Y_{sd}=\sqrt{\sum_{i=1}^{n}{\left(Y_{i}-Y_{mean}\right)}^{2}/n}$$

(14) $$F8:Z_{sd}=\sqrt{\sum_{i=1}^{n}{\left(Z_{i}-Z_{mean}\right)}^{2}/n}$$

where *n* denotes the number of points in the point cloud, $X_{i}$ denotes the coordinate value of the *i*-th point on the *x*-axis, $Y_{i}$ is the coordinate value of the ith point on the *y*-axis, and $Z_{i}$ represents the coordinate value of the *i*-th point on the *x*-axis.

The amplitude of the reflected radar echo signal determines the intensity of the target point cloud’s reflection. Radar Cross Section (RCS) is often utilized to characterize the echo strength of an object under the illumination of radar waves. The value of RCS is influenced by the size of the object. The RCS is greater, and the reflection intensity is higher in the human thoracic cavity due to the larger reflection area. Because the reflection intensity distribution of different postures is different, the center coordinates of the reflection intensity in different coordinate dimensions of the point cloud have been used as features, which are represented by $X_{c}$, $Y_{c}$, and $Z_{c}$ respectively. The calculation formula is:

(15) $${F9:X}_{c}=\frac{\sum_{i}^{n}X_{i}*SNR_{i}}{\sum_{i}^{n}SNR_{i}}$$

(16) $$F10:Y_{c}=\frac{\sum_{i}^{n}Y_{i}*SNR_{i}}{\sum_{i}^{n}SNR_{i}}$$

(17) $$F11:Z_{c}=\frac{\sum_{i}^{n}Z_{i}*SNR_{i}}{\sum_{i}^{n}SNR_{i}}$$

where $SNR_{i}$ represents the signal-to-noise ratio of the *i*-th point.

(2) Feature selection: Among the twelve features extracted from the point cloud data, not all of them can achieve the optimal classification of the target posture, and the effectiveness of point cloud data classification is related to the contribution of the feature to the classification. To omit unimportant features and improve the efficiency of classification, it is necessary to rank the importance of features.

Random forest algorithms can achieve feature importance ranking. The algorithm consists of multiple decision trees. The importance order is based on the contribution made by the feature in each decision tree. The calculation method of the contribution is to solve the difference of the Gini index before and after the branch of the feature on a certain node. The same method is applied to other features, and finally, the change value of a certain characteristic Gini index is divided by the change value of all the characteristic Gini indices to obtain the normalized contribution of the feature, the features are sorted based on the size of the contribution [37]. The formula for calculating the Gini index of the *i*-th tree node *q* is as follows:

(18) $${Gini}_{q}^{\left(i\right)}=1-\sum_{c=1}^{m}{\left(p_{qc}^{\left(i\right)}\right)}^{2}$$

where *m* represents the number of categories, and $p_{qc}^{\left(i\right)}$ represents the proportion of category c in node *q* on the *i*-th tree.

The importance of feature *j* in the node q of the *i*-th tree ${VIM}_{jq}^{\left(Gini)(i\right)}$, that is, the change of the Gini index before and after the branch of node *q*. The calculation formula is expressed as:

(19) $${VIM}_{jq}^{\left(Gini\right)\left(i\right)}={Gini}_{q}^{\left(i\right)}-{Gini}_{e}^{\left(i\right)}-{Gini}_{r}^{\left(i\right)}$$

where ${Gini}_{e}^{\left(i\right)}$ and ${Gini}_{r}^{\left(i\right)}$ denote the Gini indices of the two new nodes *e* and *r* after branching, respectively.

When there are *L* decision trees in the random forest and the node where feature *j* appears in decision tree *i* is set to *Q*, and then the importance of feature *j* can be expressed as:

(20) $${VIM}_{j}^{\left(Gini\right)}=\sum_{i=1}^{L}\sum_{q\in Q}{VIM}_{jq}^{\left(Gini\right)\left(i\right)}$$

Normalize all feature importance scores to obtain:

(21) $${VIM}_{j}=\frac{{VIM}_{j}^{\left(Gini\right)}}{\sum_{j}^{J}{VIM}_{j}^{\left(Gini\right)}}$$

where J represents the total number of features.

**Figure 9:**
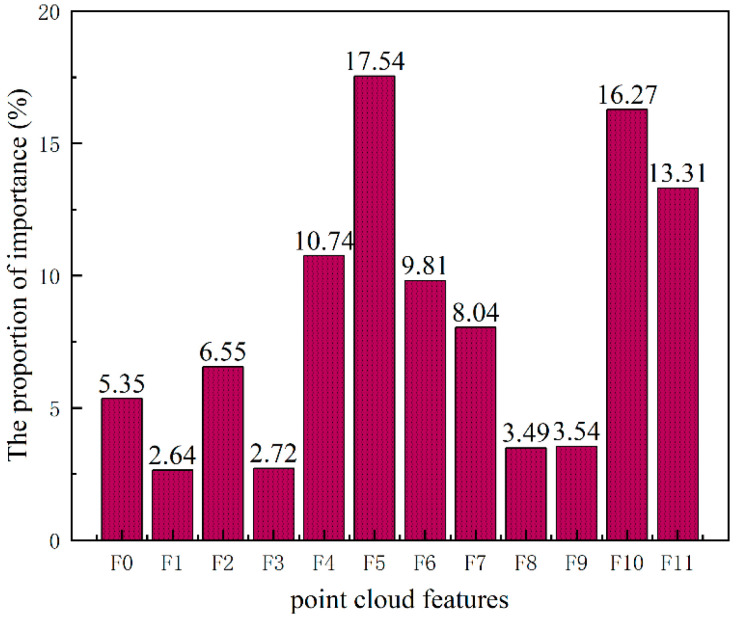
Ranking the importance of extracted point cloud features.

Python was used to import the calculated point cloud feature data into the defined random forest classifier and set 100 decision trees in the random forest model. The order of importance of all point cloud features is shown in Figure 9. The abscissa is the point cloud feature defined above, and the ordinate represents the importance of the feature. In this paper, six features with high importance are selected from the extracted features as classification features. It can be seen from the figure that F4, F5, F6, F7, F10, and F11 account for a sizeable proportion. From the point of view of physical significance, different postures are different in height, so the mean value of height direction is the most beneficial to distinguish postures. The body centroid coordinates of different postures are different in the width direction, so the mean value of the width direction is also useful to distinguish postures. In the direction of length, however, the positions of all gestures are constant, so the mean value in the direction of length does not change significantly and it is difficult to distinguish postures. There is a large gap between hands up and other postures in the direction of length and width, so it is reasonable to choose the standard deviation of length and width as the distinguishing standard. From the perspective of body reflection intensity, the positions of the main body parts (chest) in width and height are different in various postures, which will cause different central coordinates of reflection intensity in the direction of width and height, while the central coordinates of reflection intensity in the direction of length do not change significantly. Therefore, the central coordinates of reflection intensity in the direction of width and height are also important features to distinguish postures. Combining the result of the random forest feature importance ranking and the physical significance perspective of the features, these six features were selected to classify the postures.

### 3.3. Machine Learning Model

Various machine learning algorithms are not inherently good or bad, and the focus is to evaluate the accurate performance of different machine learning models and determine the most accurate classification model when faced with complex application problems. This paper selects six different machine learning models which work differently, which provide an opportunity to determine the best model for millimetre-wave point cloud postures classification. A brief introduction of all adopted machine learning models is given below.

(1) KNN: K-nearest neighbor is a non-parametric learning method. When a new sample is input, the algorithm can find the K training samples that are most similar to the new sample, so the adjustable parameters of KNN are only K values. By calculating the Euclidean distance or Manhattan distance between samples as the dissimilarity index of each sample.
(2) GP: The probabilistic-based parameter-free model for regression and classification problems. Its principle is based on Bayesian inference, which treats the input data as random variables and models the output data as Gaussian distributions. The algorithm is based on probabilistic and kernel functions, which are used to model correlations between input data points and to make predictions using Bayesian inference. It is suitable for regression and classification problems and provides predictions with confidence.
(3) SVM: The support vector machine is a classic supervised learning algorithm. Around the concept of “margin”, either side of the hyperplane separates two data classes, so SVM is a binary classification algorithm, as well as multiple binary classification problems, can be constructed to solve the multi-classification problem. Because of its robustness in multiple application types, it is regarded as a must-try method [38].
(4) MLP: The multi-layer perceptron is a forward-structured artificial neural network, consisting of an input layer, hidden layer, and output layer. Feature data has been passed from the input layer to the hidden layer, which implements the nonlinear mapping to the input space, as well as the output layer implements classification. It is noteworthy that features can be classified even with only one hidden layer because enough units are included in the hidden layer.
(5) NB: The Bayes theorem and the premise of feature condition independence underpin the Naive Bayes classification algorithm. The idea is to use the prior probability to calculate the posterior probability that a variable belongs to a certain category. The algorithm is also a type of supervised learning.
(6) GB: Gradient boosting is an efficient ensemble learning algorithm based on the lifting principle. The algorithm continuously iterates through a weak prediction model composed of decision trees to train a strong prediction model in a way that minimizes the error of the previous round [39]. It can handle large datasets with high accuracy but is slower to train due to the sequential nature of gradient boosting.

### 3.4. Multi-Class Evaluation Index

The purpose of this research is to comprehensively evaluate the performance of different machine learning models for the classification of six human postures. To achieve this purpose, traditional performance metrics are used: precision, recall, and F1 score. For classification problems, consistency in classification refers to the agreement between model predictions and actual classifications [40]. In the background of FMCW millimetre-wave human posture point cloud classification performance evaluation, the Kappa index performance index is introduced for consistency check, because the Kappa index contains the relationship between prediction accuracy and actual accuracy, two of the most important indicators. Therefore, it is of certain significance to introduce the Kappa index as an evaluation index for the classification performance of machine learning models. Meanwhile, in this research, there are 100 frames of point cloud data for each posture, and the number of target points in each frame of point cloud data is different, so it is inevitable to cause data imbalance in the process of data set division, and Kappa index can weaken the influence of unbalanced data on classification results. Furthermore, the classification outputs of each machine learning model for individual postures are visualized using ROC curves. Since the calculation of the Kappa index is based on a confusion matrix, this paper generates a corresponding confusion matrix for six machine learning models, respectively, to verify whether the introduced Kappa index can be used as an evaluation index for the performance of machine learning models. The calculation formula of each performance index is expressed as:

(22) $$A=\frac{TP+TN}{TP+TN+FP+FN}$$

(23) $$P=\frac{TP}{TP+FP}$$

(24) $$R=\frac{TP}{TP+FN}$$

(25) $$F1=\frac{2\times P\times R}{P+R}$$

(26) $$K=\frac{A-E}{1-E}$$

where A represents accuracy, P represents precision, R represents recall, F1 and K represent *F*1 score and Kappa index, respectively, TP is the number of predicted positives and actual positives, FP is the number of predicted positives and actual negatives, FN is the number of predicted negatives and actual positives, TN is the predicted negatives and the actual number of negative examples. E represents the expected accuracy, which is defined as the expected accuracy of the classifier based on the confusion matrix, expressed mathematically as:

(27) $$E=\frac{\left(TP+FN\right)\left(TP+FP\right)+\left(TN+FN\right)\left(TN+FP\right)}{{\left(TP+TN+FP+FN\right)}^{2}}$$

The accuracy rate represents the ratio of correctly recognized postures to the total number of postures. While the accuracy rate can judge the overall correct rate, it is not a perfect indicator in the case of imbalanced samples. Precision and recall, commonly used for classification evaluation, may ignore sample imbalances. Precision represents the probability that each recognized posture is correct, and recall represents the probability that a certain posture is recognized correctly. It can be seen from the definition that the two indicators are a contradiction, and the F1 score indicator is a combination of precision and recall, which can evaluate a classifier more comprehensively.

In the actual classification process, the uneven number of samples in each category would cause the model to bias the large category as well as give up the small category, particularly when in the face of multi-classification problems. The more imbalanced the confusion matrix is, the higher the *E* value is, the lower the *K* value is, and the model with significant bias can be evaluated, according to Kappa’s calculation formula. Assigns labels to different kappa ranges, as illustrated in Table 3 (see [41] for details).

Confusion matrices are examples of actual and predicted values used in the proposed model to visualize the performance of the machine learning classifier process. The deeper the color depth of the diagonal line, the higher the recognition accuracy.

The ROC curve has also been regarded as the receiver operating characteristic curve. It is a diagram that could be used to evaluate, represent, as well as select forecasting systems. The curve has two parameter values, the true positive rate (TPR) and the false positive rate (FPR), which are expressed mathematically as:

(28) $$TPR=\frac{TP}{TP+FN}$$

(29) $$FPR=\frac{FP}{FP+TN}$$

## 4. Results and Discussions

Classifications are conducted based on the research method in Section 3. All adopted machine learning model parameters are shown in Table 4, and more details given in the table can be found in Scikit-Learn, a Python-based machine learning library [42].

**Figure 10:**
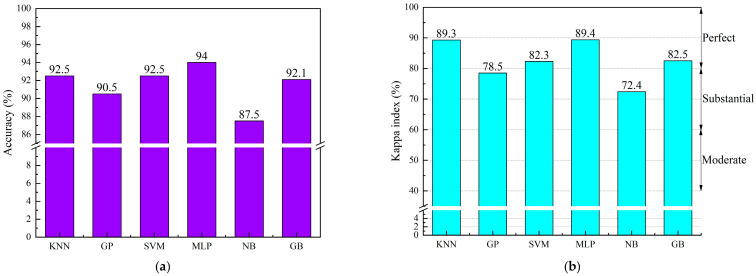
Performance of six machine learning models on Accuracy and Kappa index. (**a**) The accuracy of various machine learning models; (**b**) The Kappa index of various machine learning models.

For the data set division of point cloud feature data, this study randomly divided six hundred sets of point cloud data into the training set and testing set according to the ratio of 8:2, and input them into the machine learning model. To ensure the reliability of the results, 5-fold cross-validation was used to analyze the accuracy and Kappa index. Figure 10 presents A and K for all adopted machine learning models in the form of a bar graph. It can be seen from Figure 10a that MLP has the highest accuracy, reaching 94%, followed by KNN, SVM, GB, and the three models are close to each other, while GP and NB have poor accuracy, respectively only 90.5% and 87.5%. As can be seen from Figure 10b, except that KNN and SVM have different trends in accuracy, other Kappa indexes are consistent with accuracy. MLP has the highest K value, followed by KNN. Among them, NB has the lowest K value and its accuracy rate is also the lowest, which can be proved that NB is not suitable to be used in this paper’s dataset for classification.

**Figure 11:**
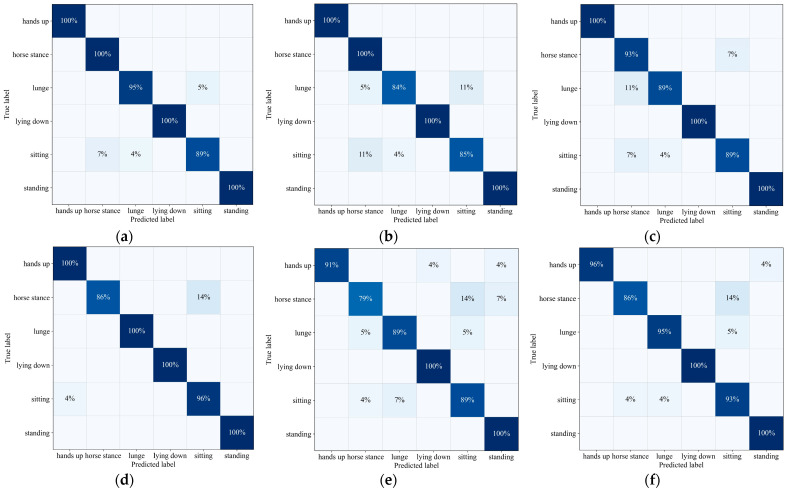
Confusion matrix for all classification models. (**a**) KNN; (**b**) GP; (**c**) SVM; (**d**) MLP; (**e**) NB; (**f**) GB.

In the case of multi-classification, a confusion matrix can be used to represent the indicator of the model performance, where the horizontal direction is the predicted label, and the vertical direction is the true label. The accuracy can be understood as the sum of the diagonals divided by the sum of the entire confusion matrix data. Therefore, the larger the diagonal data, the smaller the off-diagonal data, and the higher the recognition accuracy. The confusion matrix data for this study is shown in Figure 11. It is obvious from the confusion matrix that MLP and KNN have higher accuracy. This is consistent with the conclusion drawn by the Kappa index. The accuracy of the machine learning model evaluated by the Kappa index was verified. Among them, the accuracy rate of MLP’s recognition of horse stance is only 86%, and there is a 14% probability that it is wrong to think that it is sitting posture, while the KNN’s recognition rate of sitting posture is not high, with an accuracy of 89%, 7% probability of wrongly thinking that it is horse stance and 4% probability that it is wrongly regarded as lunge. This may be due to the fact that the point cloud shapes of horse stance and sitting are somewhat similar, and the main difference is in the height of the human posture, which is the reason sitting and horse stance are easily confused. From the six algorithms, it can be found that the recognition accuracy is 100% for both lying and standing postures, which also proves the validity of the data of the point clouds generated in this paper.

**Figure 12:**
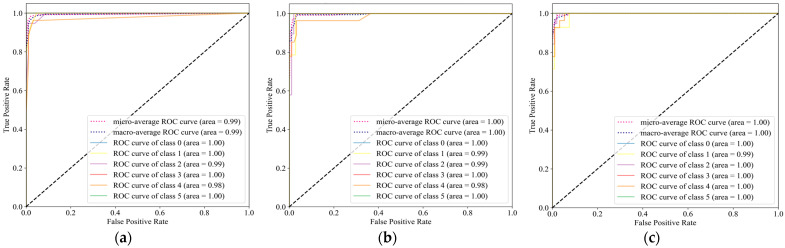

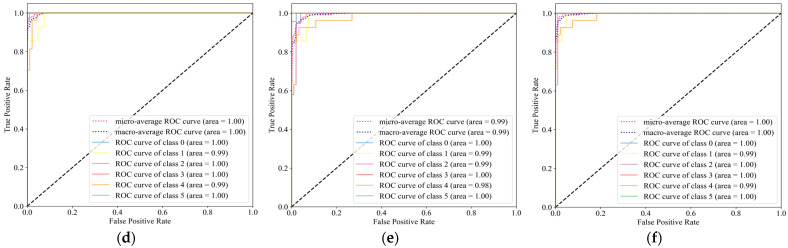
ROC curves of all classification models. (**a**) KNN; (**b**) GP; (**c**) SVM; (**d**) MLP; (**e**) NB; (**f**) GB.

The ROC curve is drawn with different thresholds and is based on the confusion matrix, with TPR and FPR as the axes. In general, the curve’s turning point is near (0, 1)—the upper left corner of the coordinates—the classifier’s classification performance. AUC refers to the area under the ROC curve and is often used as an indicator of the model’s strength or weakness. The value range is (0.5–1), with a bigger value indicating a stronger categorization effect. The macro average is to average these area values, and the micro average needs to consider the values of each dimension. Figure 12 shows that the classification performance of SVM, MLP, and GB is slightly better. The labels [0, 1, 2, 3, 4, 5] in the figure correspond to hands up, horse stance, lunges, lying down, sitting, and standing, respectively. From the area, it can be seen that the values of horse stance and sitting are relatively small.

**Figure 13:**
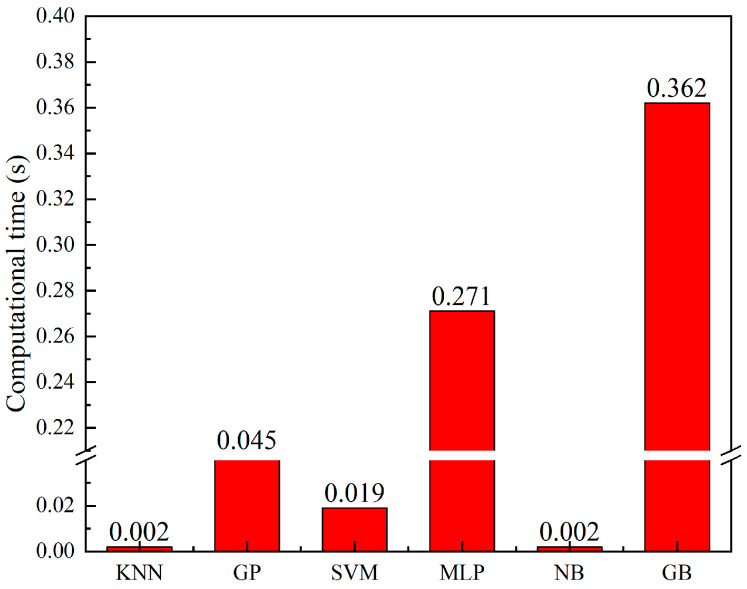
The training time of the algorithm.

Combining the accuracy, precision, recall, F1 score, Kappa value, confusion matrix, and ROC curve, it can be concluded that MLP is the classification model with the best comprehensive performance for the human posture point cloud dataset, and KNN is very stable in many indicators. However, the calculation time of the model is also one of the indicators that cannot be ignored. Figure 13 shows the training time comparison of the six models. It is evident from the figure that MLP and GB have longer computational time, while KNN and NB have the shortest computational time. The results of this training time are reasonable through the principles of the model. KNN belongs to lazy learning, which takes almost no training time because training examples are simply stored. Naive Bayesian models train fast because only one data pass is required to compute the frequency or normal probability density function. These models train orders of magnitude faster than neural network models. Gradient boosting requires constant iterations which makes its training slow.

Table 5 shows the performance of the six classification models in terms of P (precision), R (recall) and F1 (F1 score). It can be easily seen that the recognition accuracy of MLP in the three postures of the lunge, sitting, and standing is higher than other models, and KNN and SVM have the highest recognition accuracy in the hands-up posture, reaching 100%. Lying posture is recognized well in all six machine learning models, while the horse stance has the worst recognition effect among the six models. The probable reason is that the lying posture is different from other postures, with the highest degree of discrimination, while the horse stance is easily confused with the lunge and sitting postures, and the degree of discrimination is low.

## 5. Conclusions

This paper demonstrated how human posture characteristics information can be measured using FMCW Millimetre-wave radar as well as how to apply machine learning to develop a trained model having the ability to identify the human postures from the point cloud generated. The experimental study shows that FMCW millimetre-wave radar can measure the range and angle of human postures with high accuracy. The point cloud is generated from the measured feature data of human posture, which serves as the initial dataset for training machine learning models to effectively recognize human postures with new FMCW measurements. Furthermore, the comprehensive performance of different human posture classification models under the background of FMCW Millimetre-wave radar is compared and evaluated. The data input into machine learning is optimized and the dynamic and static features of human posture are integrated to make the outline of human posture in the data clearer. To show it more intuitively, the data is generated into point clouds. The clustering technique (DBSCAN algorithm) is introduced to realize the grouping of objects from the generated point cloud data. Random Forest algorithm is applied to generate feature importance ranking.

What is noteworthy is that the selection of the optimal machine learning model from the analysis is not one-size-fits-all, especially for a specific problem such as human posture classification. The neural network-based MLP method outperforms other machine learning approaches in terms of recognition accuracy, despite requiring more training time. However, it is found that in our experimental results that the NB model has the worst performance in accuracy under the given conditions.

Based on the proposed method and analysis of the results, future research can focus on increasing the number of trained models or combining the best two models in this classification, such as MLP and KNN, to further improve the accuracy of human posture classification.

## Figures and Tables

**Table 1:** Radar specific configuration.

Parameter	Description
Start frequency	60 GHz
Bandwidth	3.92 GHz
Sampling frequency	2200 ksps
Frequency slope	98 MHz/μs
Frame rate	5 fps
ADC Samples	64
Number of Chirps per frame	200

**Table 2:** Twelve proposed features and brief descriptions.

Serial Number	Symbol	Explanation
F0	*L*	The length of human 3D point clouds
F1	*W*	The width of human 3D point clouds
F2	*H*	The height of human 3D point clouds
F3	*X_mean_*	The mean value of human 3D point clouds in the length direction
F4	*Y_mean_*	The mean value of human 3D point clouds in the width direction
F5	*Z_mean_*	The mean of human 3D point clouds in the height direction
F6	*X_sd_*	The standard deviation of human 3D point clouds in the length direction
F7	*Y_sd_*	The standard deviation of human 3D point clouds in the width direction
F8	*Z_sd_*	The standard deviation of human 3D point clouds in the height direction
F9	*Xc*	The center coordinate of the reflection intensity of human 3D point clouds in the length direction
F10	*Yc*	The center coordinate of the reflection intensity of human 3D point clouds in the width direction
F11	*Zc*	The center coordinate of the reflection intensity of human 3D point clouds in the height direction

**Table 3:** Labels corresponding to different kappa indexes.

Kappa Index (%)	Label
Less than 0	Poor
0–20	Slight
21–40	Fair
41–60	Moderate
61–80	Substantial
81–100	Nearly perfect

**Table 4:** Machine learning parameters used.

ML Model	Parameter Detail
KNN	n_neighbors = 5, weights = ‘uniform’, algorithm = ‘auto’
GP	kernel = 1.0 ∗ rbf(1.0), random_state = 0
SVM	C = 33, kernel = ‘rbf’
MLP	hidden_layer_sizes = (175), activation = ‘relu’, solver = ‘lbfgs’
NB	priors = None
GB	Loss = deviance, learning_rate = 0.1, n_estimators = 100

**Table 5:** Evaluate 80% of the training model dataset on the remaining 20% of the testing dataset.

Posture	KNN			GP			SVM			MLP			NB			GB		
	P(%)	R(%)	F1(%)	P(%)	R(%)	F1(%)	P(%)	R(%)	F1(%)	P(%)	R(%)	F1(%)	P(%)	R(%)	F1(%)	P(%)	R(%)	F1(%)
hands up	100	100	100	100	100	100	100	100	100	96	100	98	100	91	95	100	96	98
horse stance	88	100	93	78	100	88	76	93	84	100	86	92	85	79	81	92	86	89
lunge	95	95	95	94	84	89	94	89	92	100	100	100	89	89	89	95	95	95
lying down	100	100	100	100	100	100	100	100	100	100	100	100	95	100	98	100	100	100
sitting	96	89	93	92	85	88	96	89	92	93	96	95	89	89	89	89	93	91
standing	100	100	100	100	100	100	100	100	100	100	100	100	89	100	94	94	100	97

## Data Availability

The data presented in this study are available upon request from the corresponding author.
